# *Streptococcus thermophilus*: A Source of Postbiotics Displaying Anti-Inflammatory Effects in THP 1 Macrophages

**DOI:** 10.3390/molecules29071552

**Published:** 2024-03-30

**Authors:** Rania Allouche, Zeeshan Hafeez, Annie Dary-Mourot, Magali Genay, Laurent Miclo

**Affiliations:** CALBINOTOX, Université de Lorraine, F-54000 Nancy, France; allouche.rania@yahoo.fr (R.A.); annie.dary@univ-lorraine.fr (A.D.-M.); magali.genay@univ-lorraine.fr (M.G.); laurent.miclo@univ-lorraine.fr (L.M.)

**Keywords:** *S. thermophilus*, postbiotics, intracellular proteins, pancreatic proteases, anti-inflammatory, THP-1 macrophages

## Abstract

In addition to traditional use in fermented dairy products, *S. thermophilus* also exhibits anti-inflammatory properties both in live and heat-inactivated form. Recent studies have highlighted that some hydrolysates from surface proteins of *S. thermophilus* could be responsible partially for overall anti-inflammatory activity of this bacterium. It was hypothesized that anti-inflammatory activity could also be attributed to peptides resulting from the digestion of intracellular proteins of *S. thermophilus*. Therefore, total intracellular proteins (TIP) from two phenotypically different strains, LMD-9 and CNRZ-21N, were recovered by sonication followed by ammonium sulphate precipitation. The molecular masses of the TIP of both strains were very close to each other as observed by SDS-PAGE. The TIP were fractionated by size exclusion fast protein liquid chromatography to obtain a 3–10 kDa intracellular protein (IP) fraction, which was then hydrolysed with pancreatic enzyme preparation, Corolase PP. The hydrolysed IP fraction from each strain exhibited anti-inflammatory activity by modulating pro-inflammatory mediators, particularly IL-1β in LPS-stimulated THP-1 macrophages. However, a decrease in IL-8 secretion was only observed with hydrolysed IP fraction from CNRZ-21N, indicating that strain could be an important parameter in obtaining active hydrolysates. Results showed that peptides from the 3–10 kDa IP fraction of *S. thermophilus* could therefore be considered as postbiotics with potential beneficial effects on human health. Thus, it can be used as a promising bioactive ingredient for the development of functional foods to prevent low-grade inflammation.

## 1. Introduction

*Streptococcus thermophilus*, a lactic acid bacterium regarded as ‘Generally Recognized as Safe’ (GRAS) by the FDA and ‘Qualified Presumption of Safety’ (QPS) by EFSA, is widely used in the food industry. Indeed, *S. thermophilus* is one of the main starter bacteria of yogurt, and it could also be used to prevent or delay onset of certain human pathologies such as chronic gastritis and diarrhoea [1]. These health effects are consistent with the fact that this bacterium is considered a probiotic by many authors. Nevertheless, some of them are questioning the use of the term probiotic for *S. thermophilus* species due to its sensitivity when transiting through the human digestive environment. Thus, Martinović et al. (2020) wondered about the methods used to evaluate the survival of *S. thermophilus* in the gastrointestinal tract, reporting that they could not be reliable because the identification of this bacterium from stool is often confused, in most studies, with a common human commensal *Streptococcus salivarius*, a species that is phylogenetically close to *S. thermophilus* [2]. Since bacterial viability is not always a requirement for a probiotic to exert a beneficial effect on human health, the concept of postbiotics has emerged in recent years [3]. The term “postbiotic” was first consensually defined in 2021 by the International Scientific Association of Probiotics and Prebiotics (ISAPP) as “a preparation of inanimate microorganisms and/or their components that confers a health benefit on the host”. According to this definition, a postbiotic requires the presence of entire inactivated microbial cells or cell components with or without cell metabolic products [4]. Postbiotics can be soluble factors secreted by live bacteria or released after bacterial lysis like proteins, peptides, teichoic acids, peptidoglycan-derived muropeptides, exopolysaccharides or organic acids [5,6]. One of the most commonly used approaches to recover certain classes of postbiotics is the cell disruption that employs heating, irradiation, the use of high pressures as well as sonication techniques [5,7,8].

Beneficial health effects of various non-viable probiotics and/or lactic acid bacteria have been demonstrated in different studies. For example, oral intake of heat-inactivated *Bifidobacterium longum* BR-108 significantly decreased the body weight gain and blood glucose levels in obese diabetic mice [9]. Orally administered heat-killed *Lactobacillus gasseri* TMC0356 enhanced the natural defence mechanisms against pathogenic infections in elderly subjects [10]. Similarly, a lactic acid bacterial strain *Lacticaseibacillus paracasei* MCC1849 displayed immuno-modulatory effects in humans in non-viable heat-killed form [11]. Likewise, a bacteria-free culture media derived from *Bifidobacterium breve* C50 and *S. thermophilus* 065 improved T helper 1 immune response and intestinal barrier in mice [12]. Furthermore, previous in vitro and in vivo studies have shown that *S. thermophilus* exhibits anti-inflammatory properties not only in live form but also in a heat-inactivated form [3,13,14,15]. Additionally, postbiotics could also be released after cell lysis during gastrointestinal passage. It has been reported that a part of *Lactococcus lactis* cells die while passing through the gastrointestinal tract of rats, and the majority of dead cells appears to undergo rapid lysis, particularly in the duodenum. This last phenomenon was more pronounced when a large population of *L. lactis* was mixed with food compared to one containing normal level of bacterial population [16]. Therefore, it can be assumed that a part of *S. thermophilus* cells can undergo lysis during gastrointestinal passage, leading to the release of intracellular proteins. The latter, in turn, could serve as substrates for digestive proteases and thereby generate peptides, potentially postbiotics. Indeed, in our previous studies, we recovered the surface proteins from two different *S. thermophilus* strains by enzymatic shaving. The surface proteins were hydrolysed by the digestive enzyme trypsin to generate peptide hydrolysates which exhibited anti-inflammatory activity in two cell models: HT-29 cells and THP-1 macrophages [17,18]. Thus, it was suggested that peptide(s) from these hydrolysates could be responsible for at least in part of the overall anti-inflammatory activity of *S. thermophilus*. However, few peptides contained in these hydrolysates corresponded to cytoplasmic proteins, which could result from lysis of few bacterial cells or from moonlighting proteins [17]. So, both extracellular and intracellular bacterial proteins might be a source of anti-inflammatory peptides. Consequently, it could be hypothesized that the anti-inflammatory activity could also be attributed to peptides derived from the digestion of intracellular proteins of *S. thermophilus*.

Therefore, the aim of this study was to investigate whether hydrolysates obtained after hydrolysis by pancreatic proteases of intracellular proteins of two phenotypically different strains of *S. thermophilus,* LMD-9 and CNRZ-21N, could be involved in anti-inflammatory properties. The potential anti-inflammatory effects of hydrolysates were evaluated in human THP-1 macrophages.

## 2. Results and Discussion

### 2.1. Purification of Total Intracellular Proteins from S. thermophilus Strains

#### 2.1.1. Characterization of Total Intracellular Proteins by Sodium Dodecyl Sulphate-Polyacrylamide Gel Electrophoresis

**Figure 1:**
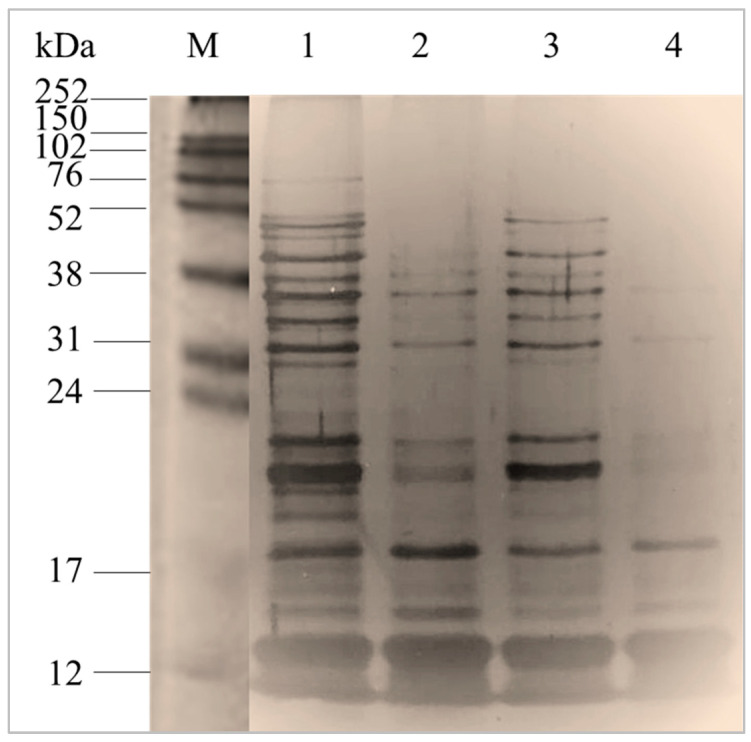
Electrophoretic profiles from SDS-PAGE analysis of total intracellular proteins of *S. thermophilus* LMD-9 (TIP-9) and CNRZ-21N (TIP-21) obtained after cell lysis followed by ammonium sulphate precipitation. Quantities of 20 µg and 10 µg of TIP-9 (lanes 1 and 3) and TIP-21 (lanes 2 and 4) were loaded onto 15% separation gel. Proteins were stained by silver nitrate. M: molecular weight marker.

Sodium dodecyl sulphate-polyacrylamide gel electrophoresis (SDS-PAGE) was performed to characterise the molecular weight profile of the total intracellular proteins (TIP) of *S. thermophilus* LMD-9 (TIP-9) and CNRZ-21N (TIP-21) strains obtained after cell lysis followed by ammonium sulphate precipitation. The electrophoretic profiles of TIP of *S. thermophilus* strains were very close to each other (Figure 1). However, some protein bands seemed to lack in the TIP profile of CNRZ-21N strain compared to that of LMD-9. A recent study that analysed 23 genomes of this bacterium showed that most of the *S. thermophilus* strains could be classified into two groups according to the genome size (greater or not than 1.83 Mbp) linked to events of the loss or gain of genes [19]. According to this classification, the LMD-9 strain belongs to group A with a genome greater than 1.83 Mbp, while the CNRZ-21N strain can be classified in group B since its genome size is of 1,819,203 bp (unpublished result). Moreover, all strains belonging to the group B lack the *prtS* gene coding the cell-wall protease PrtS, which is also the case for the CNRZ-21N strain.

#### 2.1.2. Fractionation of Total Intracellular Proteins by Size Exclusion Fast Protein Liquid Chromatography

**Figure 2:**
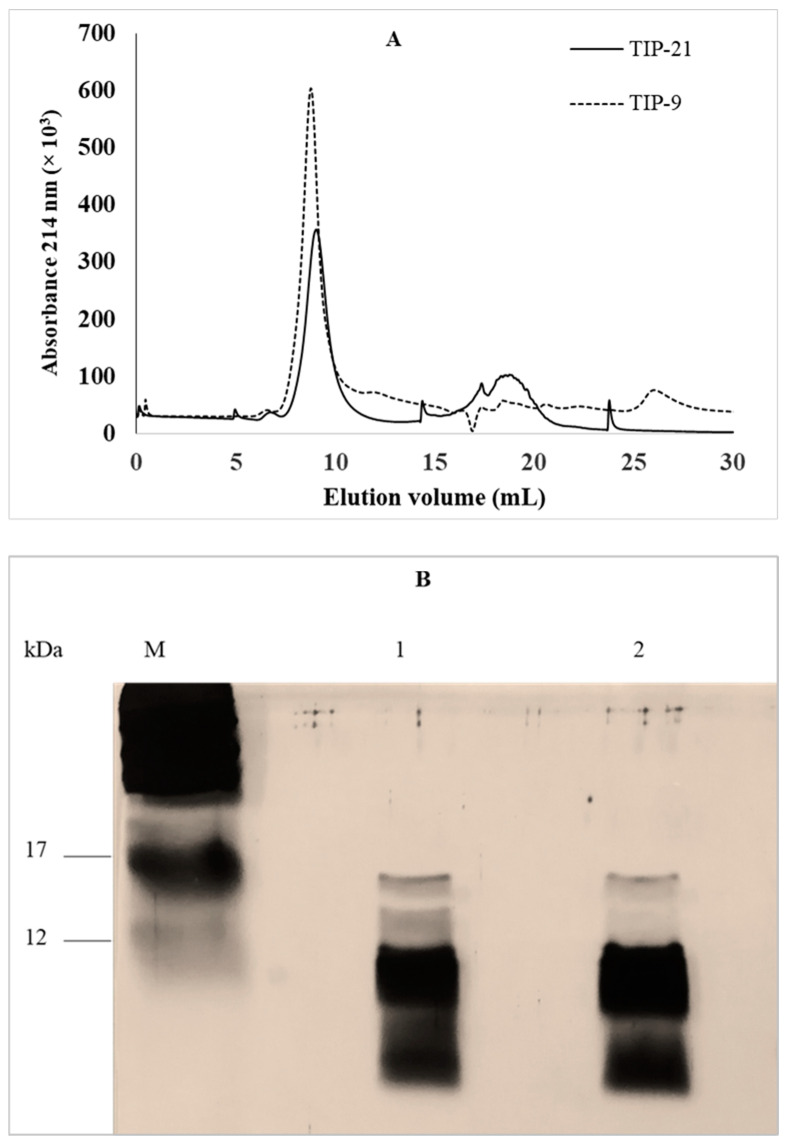
(**A**) Chromatographic profiles obtained by SE-FPLC of TIP-9 (dotted line) and TIP-21 (continuous line) recovered after cell lysis followed by ammonium sulphate precipitation of TIP of *S. thermophilus* LMD-9 and CNRZ-21N, respectively. TIP-9 and TIP-21 were injected onto a Superdex™ Peptide HR 10/30 column and eluted at a flow rate of 250 µL/min using a solvent containing 30% (*v*/*v*) acetonitrile and 0.1% (*v*/*v*) trifluoroacetic acid in milli-Q water. The eluted proteins were detected by absorbance measurement at 214 nm. IP-9 and IP-21 fractions corresponding to molecular mass of 3–10 kDa were collected between 7 and 11 mL from TIP-9 and TIP-21, respectively. (**B**) Electrophoretic profiles from SDS-PAGE analysis of IP-9 (lane 1) and IP-21 (lane 2) fractions. Both IP-9 and IP-21 fractions were loaded at 5 µg onto 20% separation gel. Proteins were stained by silver nitrate. M: molecular weight marker.

*S. thermophilus* has a significant impact on the texture of yogurts as this bacterium produces exopolysaccharides [20]. Among 23 strains with different genomes, including the LMD-9 strain, all possess a cluster of genes involved in the synthesis of these compounds [19]. As exopolysaccharides are known to possess anti-inflammatory activity [21], therefore, these molecules should be removed from the protein fractions which would have to be evaluated in the THP-1 macrophages model. Exopolysaccharides have a molecular mass between 58 and 180 kDa [22]. To remove them all, it was chosen to collect from TIP of both strains an intracellular protein (IP) fraction with molecular masses between 3 and 10 kDa by size exclusion fast protein liquid chromatography (SE-FPLC). A single peak with an elution volume between 7 and 11 mL was collected from the TIP-9 and TIP-21 samples (Figure 2A). The chromatographic profiles obtained for the two strains showed that there were very few proteins possessing an elution volume of less than 7 mL, whereas proteins of molecular mass up to 80 kDa were present in the TIP samples of both strains (Figure 1). An absence of solubilization in milli-Q water of part of the proteins contained in the samples before injection into the SE-FPLC column could explain this observation. As expected, the electrophoretic profile of IP fractions collected with a molecular mass between 3 and 10 kDa revealed that the proteins present in the IP fraction of *S. thermophilus* LMD-9 (IP-9) or CNRZ-21N (IP-21) were less than 17 kDa, with the majority being less than 12 kDa. Despite the strong intensity of the silver nitrate staining for the bands with a molecular mass below 12 kDa, differences were still visible between the protein profile of the IP-9 and that of the IP-21 fractions (Figure 2B).

#### 2.1.3. LC-MS Analysis of IP-9 and IP-21 Fractions

**Figure 3:**
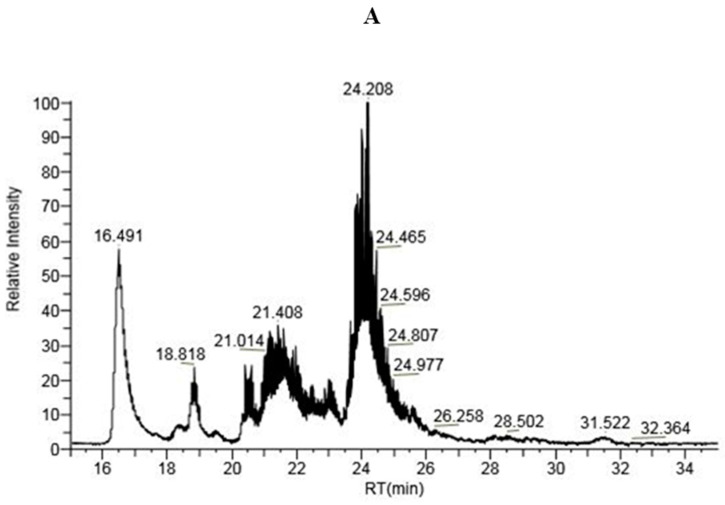

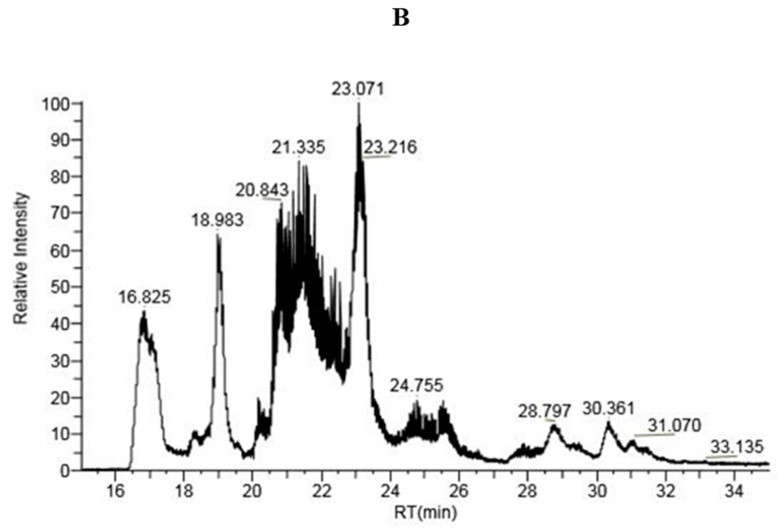
LC-MS chromatograms of intracellular protein fraction IP-9 of LMD-9 strain (**A**) and IP-21 of CNRZ-21N strain (**B**). IP fractions were obtained after fractionation by SE-FPLC of total intracellular proteins (TIP) recovered after cell lysis and ammonium sulphate precipitation.

The results obtained after the LC-MS analysis of the IP-9 and IP-21 samples showed that majority of the proteins present either in the IP-9 fraction or IP-21 fraction displayed a molecular mass between 5 and 11 kDa. This result was consistent with (i) the area of elution volume collected after fractionation by SE-FPLC which corresponded to a molecular mass of 3–10 kDa and (ii) the electrophoretic mobilities visualized in SDS-PAGE (Figure 2B). Even if the intensity of the peaks did not depend on the quantity but on the ionization of the proteins, the LC-MS chromatograms (intensity vs. retention time) obtained for IP fractions of the two strains of *S. thermophilus* showed significant differences (Figure 3). There was a high-intensity peak for a retention time of about 23 min for the CNRZ-21N strain and a mass of high-intensity peaks for retention times between 23.5 and 25.5 min for the LMD-9 strain. It was not possible to characterize the concerned proteins so far as there were many proteins of very similar molecular mass and that differences can exist in molecular mass for a same protein due to presence of mutations in the sequence between LMD-9 and CNRZ-21N strains. Nevertheless, these results confirmed that the IP fractions obtained from the TIP of both strains of *S. thermophilus* presented a different protein composition, difference which was already suggested in the analysis of the results obtained by SDS-PAGE of the same fractions.

### 2.2. Identification of Intracellular Proteins by LC-MS/MS Analysis of the Peptides Obtained after Hydrolysis of Intracellular Protein Fractions by Corolase PP

The enzyme used to hydrolyse IP fractions was Corolase PP. This enzymatic complex is a mixture of endopeptidases and exopeptidases such as trypsin, chymotrypsin, elastase and aminopeptidases, as well as carboxypeptidase A1, A2 and B [23], but it does not serve the purpose of mimicking the whole GIT conditions. The IP-9 and IP-21 fractions were hydrolysed with Corolase PP, and then the peptides present in the intracellular protein hydrolysates (named IPH-9 and IPH-21, respectively) were characterised by tandem mass spectrometry. Approximately 50 and 45 proteins for LMD-9 and CNRZ-21N strains, respectively, were identified in the IP fractions after LC-MS/MS analysis of the obtained peptides. For subsequent analysis, only proteins generating a significant number of peptides (≥10) and/or percentage coverage (≥50%) were considered, resulting in 31 proteins for both LMD-9 and CNRZ-21N strains (Table 1). Ribosomal proteins were the most abundant family of proteins highlighted in IP-9 and IP-21 fractions. In fast-growing *E. coli*, these proteins can represent up to 50% of the proteome [24]. A bacterial cell of this species can contain about 20,000 ribosomes. Depending on the organism, ribosomal proteins constitute between 30 and 70% of the total mass of a ribosome [25]. The 50S ribosomal subunit protein L24 (STER_1896) was the most abundant protein and generated a significant number of peptides, i.e., 78 and 126 for LMD-9 and CNRZ-21N strains, respectively. The putative ribosomal protein (STER_0787) and the 50S ribosomal protein L18 (STER_1891) were only identified in IPH from LMD-9 strain and generated 15 and 11 sequences, respectively. In contrast, the ribosomal protein S21 and L31 were only identified in IPH of CNRZ-21N strain with nine peptide sequences. The molecular masses of the proteins identified in the hydrolysates of the IP-9 and IP-21 fractions ranged from 4.92 to 15.52 kDa with the exception of the ribosomal proteins L6 and L2 whose molecular masses were 19.44 and 29.91 kDa, respectively. The molecular mass of ribosomal protein L2 was far outside the collection range in SE-FLPC. This protein is known to display a very strong affinity for some surfaces, especially that of silica [26]. Interactions between L2 protein and the column may have occurred, delaying its outlet and consequently increasing its elution volume.

### 2.3. Predicted Anti-Inflammatory Activity Based on In Silico Simulation

IHP-9 and IHP-21 yielded 778 and 895 peptide sequences, respectively. In silico screening of these peptide sequences indicated a significant percentage of peptides with anti-inflammatory potential (AIP). Indeed, 45.1% of peptides from LMD-9 strain (338 out of 750 sequences identified) and 52.9% of peptides from CNRZ-21N strain (460 out of 869) were predicted to display a high AIP based on database parameters (score range ≥0.468, sensitivity of 63.2% and specificity of 90.3%). A score of ≥0.468 was labelled as high-confidence AIP, while 0.468  >  score  >  0.388 was labelled as medium-confidence AIP and 0.388  >  score  >  0.342 was labelled as low-confidence AIP.

### 2.4. Anti-Inflammatory Activity of Intracellular Protein Hydrolysates IPH-9 and IPH-21

**Figure 4:**
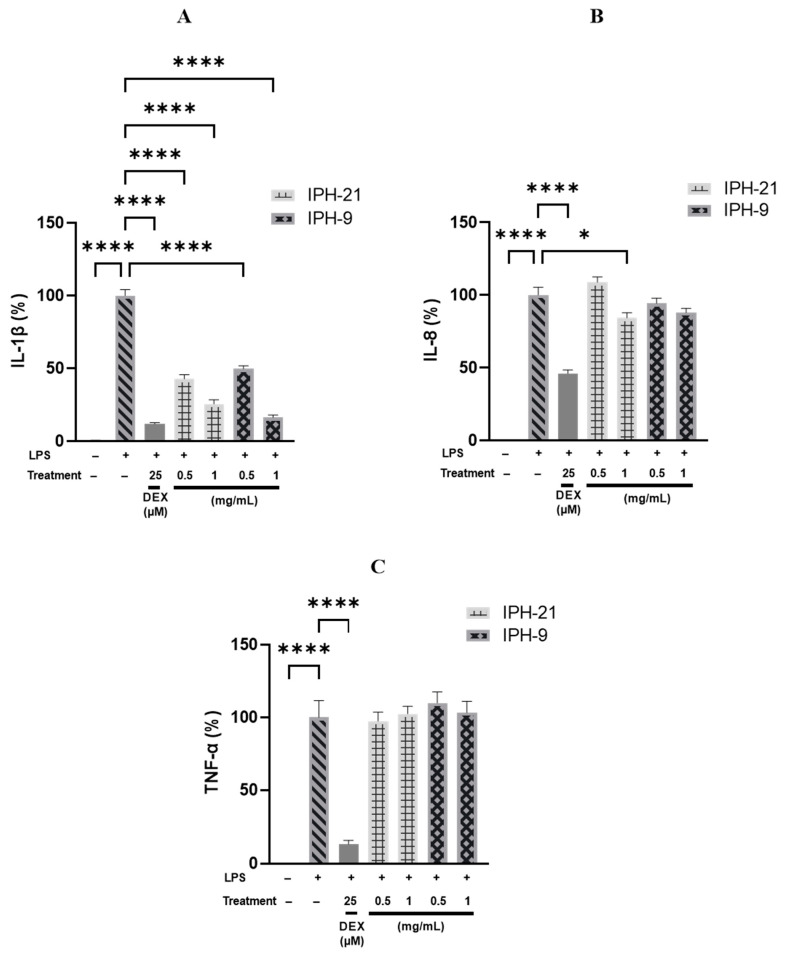
Effects of IPH-9 and IPH-21, obtained after hydrolysis by Corolase PP of IP-9 and IP-21 fractions from the *S. thermophilus* LMD-9 and CNRZ-21N strains, respectively, on the secretion of (**A**) IL-1β, (**B**) IL-8 and (**C**) TNF-α cytokines in LPS-inflamed THP-1 macrophages. Cells were co-incubated with LPS (50 ng/mL) and with or without IPH-9 or IPH-21 (0.5 or 1 mg/mL) for 3 h. The negative control (LPS−) corresponds to untreated cells. Dexamethasone (DEX) at 25 µM was used as a positive control (gray bars without pattern). IL-8 (%), TNF-α (%) and IL-1β (%) are the percentages of IL-8, TNF-α or IL-1β, respectively, secreted by cells compared to their secretions by cells treated only with LPS (100% control; gray hatch filled bars). Results are expressed as means ± SEM. * *p* < 0.05, **** *p* < 0.0001.

**Figure 5:**
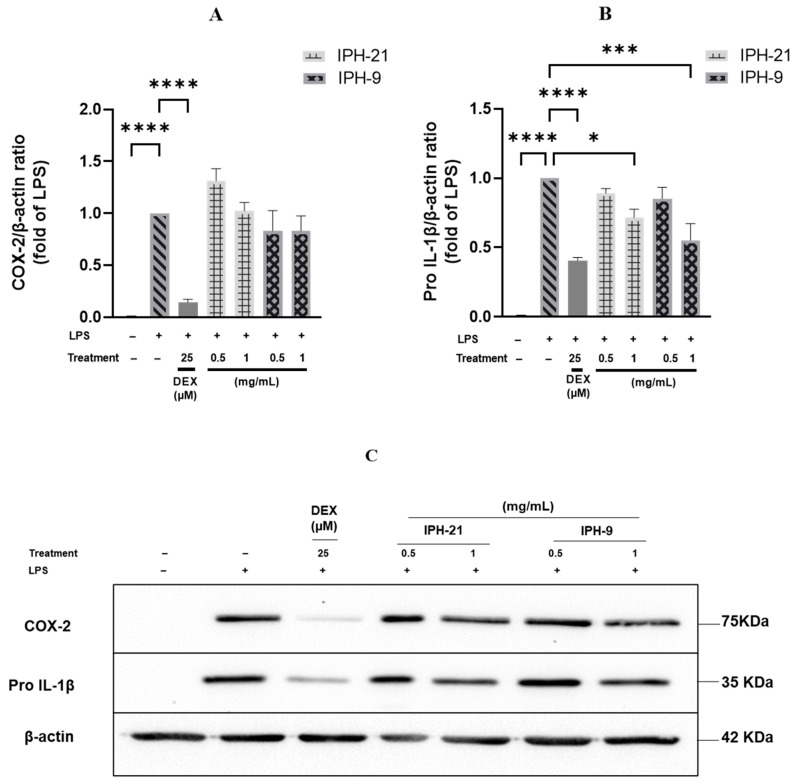
Effects of IPH-9 and IPH-21, obtained after Corolase PP hydrolysis of IP-9 and IP-21 fractions from the *S. thermophilus* LMD-9 and CNRZ-21N strain, respectively, on COX-2 and pro-IL-1β protein expression in LPS-inflamed THP-1 macrophages. (**A**–**C**) THP-1 macrophages were co-incubated with LPS (50 ng/mL) alone (gray hatch filled bars) or with LPS and IPH-9 or IPH-21 at (0.5 or 1.0 mg/mL) for 3 h. Dexamethasone (DEX) at 25 µM was used as a positive control (gray bars without pattern). Fifteen µg of each protein sample was loaded onto a 10% polyacrylamide gel containing SDS. The results were calculated as a relative intensity to the β-actin. Results are expressed as means ± SEM. * *p* < 0.05, *** *p* < 0.001, **** *p* < 0.0001.

LMD-9 and CNRZ-21N strains are phenotypically different and can be classified into two distinct *S. thermophilus* groups. The IP-9 and IP-21 fractions obtained after fractionation of TIP revealed a different protein composition in qualitative and potentially quantitative terms. The hydrolysates IPH-9 and IPH-21 obtained after Corolase PP hydrolysis of IP-9 and IP-21 fractions, respectively, displayed a different peptide composition. A significant percentage of the peptides present in IPH-9 or IPH-21 were predicted in silico to be anti-inflammatory. Hence, the anti-inflammatory activity of these hydrolysates was evaluated in vitro in THP-1 macrophages model, which is commonly used to study the action on inflammation of diverse types of drugs as well as food components [27,28]. THP-1 macrophages were co-incubated for 3 h with 0.5 or 1.0 mg/mL of IPH-9 or IPH-21 in presence of LPS. Inflammation was evaluated by measuring the secretion of pro-inflammatory cytokines TNF-α, IL-1β and IL-8 and the production of pro-IL-1β and COX-2 by THP-1 macrophages. The cell viability in the tested conditions was over 98% (*p* < 0.05);. The secretion of pro-inflammatory cytokines was significantly higher for LPS-stimulated THP-1 macrophages than for untreated cells (Figure 4), demonstrating that LPS brought THP-1 macrophages into an inflammatory state. However, co-treatment of THP-1 macrophages with LPS and IPH-9 or LPS and IPH-21 reduced IL-1β secretion in a concentration-dependent manner. IPH-9 and IPH-21 at 0.5 mg/mL decreased IL-1β secretion in LPS-stimulated THP-1 macrophages by 50.3% and 57.3%, respectively (*p* < 0.0001, Figure 4A). A greater reduction in IL-1β secretion of 83.8% and 74.6% was observed with a concentration of 1 mg/mL of IPH-9 and IPH-21, respectively (*p* < 0.0001, Figure 4A). The difference in the reduction of IL-1β secretion was not statistically significant when the same concentration of peptide hydrolysate was used regardless of the source of the IP fraction from which the hydrolysates were originated. Interestingly, difference in the decrease of IL-1β secretion between cells treated with 1 mg/mL of IPH-9 and those treated with dexamethasone, a synthetic glucocorticoid, at 25 µM was not significant. It has been shown that postbiotics from *Lacticaseibacillus rhamnosus*, *Limosilactobacillus reuteri* and *Bifidobacterium animalis* subsp. *lactis* Bb12 reduced the release of mature IL-1β by LPS-primed, cSiO_2_-stimulated macrophages [29]. Moreover, increased IL-6 mRNA quantity and decreased mRNA quantities of IL-1β, TNF and IL-10, an anti-inflammatory cytokine, were observed in the jejunum of lambs receiving *Lactiplantibacillus plantarum* RG14 postbiotics [30]. Co-treatment of THP-1 macrophages with LPS and IPH-9 at 0.5 or 1.0 mg/mL or with LPS and IPH-21 at 0.5 mg/mL did not result in a statistically significant reduction in IL-8 secretion relative to THP-1 macrophages treated only with LPS. However, IPH-21 at 1 mg/mL led to a significant decrease of IL-8 release by 15.7% (*p* < 0.05, Figure 4B). On the other hand, the secretion levels of TNF-α cytokine caused by LPS-stimulation remained unchanged regardless of the concentrations tested of IPH-9 or IPH-21 (Figure 4C). The lack of effect on the secretion of TNF-α in this cell model had already been observed with the hydrolysates of surface proteins of both LMD-9 and CNRZ-21N strains [17,18]. Similarly, it was shown that phenolic-rich extracts from common beans were unable to suppress TNF-α mRNA expression contrary to IL-1β and IL-6 in LPS-stimulated RAW 264.7 macrophages [31]. The expression of pro-IL-1β and COX-2 was determined by Western blot analysis (Figure 5). The expression level of pro-IL-1β was significantly reduced by 44.8% and 28.5% upon co-treatment of LPS-inflamed THP-1 macrophages with 1 mg/mL of IPH-9 and IPH-21, respectively, (*p* < 0.05, *p* < 0.001, Figure 5B). The expression of COX-2 remained unaffected in LPS-inflamed THP-1 macrophages regardless of the concentration used of IPH-9 or IPH-21 (Figure 5A). In contrast, dexamethasone at 25 µM upon co-incubation with LPS-inflamed THP-1 macrophages significantly reduced the COX-2 expression by 85.6% (Figure 5A). The overall results of this study showed that peptides derived from the hydrolysis by Corolase PP of the intracellular proteins with a molecular mass between 3 and 10 kDa of two phenotypically different strains of *S. thermophilus* can lead to the production of postbiotics displaying an effect on the modulation of inflammation involving in particular the IL-1β cytokine. This effect is very close to that of dexamethasone with a concentration of 1 mg/mL of hydrolysate. The action on IL-8 secretion is weaker and was only highlighted with IPH-21 obtained from the CNRZ-21N strain at a concentration of 1 mg/mL. Modulation of IL-1β may be an interesting way of action since the specific targeting of IL-1β by neutralizing antibodies seems judicious in order to improve some auto-inflammatory diseases [32]. 

The host–microbiota crosstalk is fundamental in the mechanisms regulating intestinal homeostasis. The cells of the colonic mucosa of patients with inflammatory bowel disease (IBD) secrete more pro-inflammatory cytokines than those of healthy individuals, particularly IL-1β whose concentration was nine-times higher in IBD patients [33]. The microbiota can modulate intestinal immunity by direct contact with the intestinal mucosa, by short-chain fatty acids derived from fibre metabolism or by peptides originating from the exoproteome of microbiota bacteria. The exoproteome is made up of proteins from the cellular secretion, export phenomena and cell lysis whose relative abundance depends on their stability [34]. Two peptides from microbiota bacteria, the 17-mer peptide B12 from *Bacteroides fragilis* YCH46 and the 19-mer peptide B7, with tolerogenic potential, from *Bifidobacterium longum* subsp. *longum* ATCC 15707 showed modulation of cytokine production in human lamina propria mononuclear cells. However, these two peptides decreased the secretion of anti-inflammatory cytokine IL-10 in colonic mucosal cells from patients suffering from active phase of IBD [33]. The action of peptides on severely inflamed tissues may not be sufficient. Consequently, probiotics and/or peptides derived from them may help maintain satisfactorily intestinal homeostasis in healthy patients but may be ineffective or even harmful in individuals suffering from IBD. Thus, the B7 peptide acting on the C-C chemokine receptor type 2 (CCR2) may reduce the migration of monocytes towards the intestine in healthy people, while it may be ineffective in people with IBD [33]. Peptides from the 3–10 kDa fraction of intracellular proteins of *Streptococcus thermophilus* showed anti-inflammatory activity in THP-1 cells, which can be a model mimicking macrophages as mediators of intestinal homeostasis, but this result does not prejudge the activity of these peptides in people suffering from acute phase of IBD since an opposite effect might also be demonstrated as was the case with other probiotics or postbiotics.

## 3. Materials and Methods

### 3.1. Bacterial Strains and Culture Conditions

*S. thermophilus* LMD-9 (purchased from American Type Culture Collection, Manassas, VA, USA) and CNRZ-21N (isolated from yogurt in our laboratory) were stored at −20 °C in sterile reconstituted skim milk (10%, *w*/*v*). Bacterial strains were precultured overnight in reconstituted skim milk (10%, *w*/*v*) and then cultured in LM7 broth (M17 broth supplemented with 2% lactose) as described previously by Allouche et al. [17,18]. The optical density at 650 nm (OD_650nm_) of cultures was measured to follow the bacterial growth.

### 3.2. Extraction of Total Intracellular Proteins

The TIP of *S. thermophilus* LMD-9 or CNRZ-21N strains were recovered using a sonication approach. Strains were cultured in LM17 broth with 1% preculture and incubated at 42 °C according to Allouche et al. [17,18]. Once the cultures reached an OD_650nm_ of 4 (end of exponential growth phase), cells were harvested by centrifugation at 3320× *g* for 10 min at 20 °C and washed thrice with PBS 10 mM (137 mM NaCl, 2.7 mM KCl, 10 mM Na_2_HPO_4_, 2 mM KH_2_PO_4_), pH 7.4. The washed cells were concentrated to an OD_650nm_ of 10 (final volume of 30 mL) by resuspending them in HCl 10 mM, pH 2. Then, cell suspensions were placed on ice and lysed by sonication (Branson Digital Sonifier, Hampton, NH, USA): 30 s of sonication for 10 cycles with a one-minute pause between each active cycle of 50%. The cell lysates were then separated from the cell debris by centrifugation at 3320× *g* for 10 min at 4 °C.

### 3.3. Protein Precipitation

TIP from cell lysates of both *S. thermophilus* strains were retrieved by the ammonium sulphate precipitation method. For this, the cell lysates of each strain were brought to 80% ammonium sulphate saturation by adding directly solid ammonium sulphate to these lysates. The solutions were stirred overnight at 4 °C and then centrifuged at 3320× *g* at 4 °C for one hour. The supernatants were discarded, and the resulting TIP (TIP-9 or TIP-21) pellets were redissolved in milli-Q water and conserved at −20 °C until further analysis.

### 3.4. Size-Exclusion Fast Protein Liquid Chromatography

The TIP (TIP-9 or TIP-21) solutions in water were fractionated by SE-FPLC. First, the column was calibrated using standard peptides of known molecular masses: bovine α-lactalbumin, 14,175 Da; aprotinin of bovine origin, 6500 Da; hexaGly, 306.32 Da; Gly-Gln, 203 Da; Tyr, 181.19 Da (Sigma-Aldrich, Saint Quentin Fallavier, France). The relationship between the partition coefficient Kav and the logarithm of the molecular mass of the standard peptides made it possible to calculate the elution volumes of the peptide fractions of interest. The standards or samples were injected through a valve with a 250 µL sample loop linked to a Superdex™ Peptide HR 10/30 (10 × 30 mm) analytical column (Amersham Pharmacia Biotech, Uppsala, Sweden) connected to a FPLC system (ÄKTA-FPLC, Amersham Pharmacia Biotech). Proteins were eluted at a flow rate of 250 µL/min using a solvent containing 30% (*v*/*v*) acetonitrile (ACN) and 0.1% (*v*/*v*) trifluoroacetic acid (TFA) in milli-Q water. Detection of eluted proteins was performed by absorbance measurement at 214 nm. For each strain, the fraction with a molecular mass ranging between 3 and 10 kDa, free of non-protein contaminants, was collected from TIP (TIP-9 and TIP-21) and was subsequently called the IP fraction (IP-9 and IP-21, respectively). The latter were lyophilized, weighed and stored at −20 °C until enzymatic hydrolysis or SDS-PAGE and LC-MS/MS analysis.

### 3.5. SDS-PAGE Analysis of TIP

The TIP-9 or TIP-21 retrieved by ammonium sulphate precipitation from the cell lysates of both strains, as well as the IP-9 and IP-21 fractions obtained after SE-FPLC fractionation, were resolved by SDS-PAGE. Prior to SDS-PAGE analysis, the residual ammonium sulphate present in TIP-9 or TIP-21 samples was removed by filtration using Amicon^®^ Ultra-0.5 centrifugal filter devices (Merck-Sigma, Darmstadt, Germany). Afterwards, all the protein samples were independently dissolved in sample buffer (2× Laemmli buffer; 5% ß-mercaptoethanol (*v*/*v*)) and heated for 5 min at 100 °C. Then, 10 or 20 µg of TIP-9 or TIP-21 and 5 µg of the IP fractions (IP-9 or IP-21) were loaded onto a 15% and 20% separation polyacrylamide gels, respectively. Electrophoresis was performed at a voltage of 120 V in a buffer (25 mM Tris, 192 mM glycine, 0.1% (*w*/*v*) SDS, pH 8.3) for 90 min at room temperature. After migration, proteins were visualized by silver staining, as previously described by [35,36] with some modifications. Briefly, after fixation and sensitization with 0.02% sodium thiosulfate, gels were submerged in 0.1% silver nitrate (*w*/*v*) for 45 min. The coloration was then developed with a solution containing 12 g of sodium carbonate, 2% of sodium thiosulfate and 35% formaldehyde. Proteins molecular masses were estimated using a molecular weight marker (12–225 kDa, Cytiva Rainbow™, Fisher Scientific, Illkirch, France).

### 3.6. Enzymatic Hydrolysis

IP-9 and IP-21 fractions were solubilized in 10 mM PBS (137 mM NaCl, 2.7 mM KCl, 10 mM Na_2_HPO_4_, 2 mM KH_2_PO_4_), pH 7.4 and were hydrolysed with the pancreatic-enzyme complex, Corolase PP (AB Enzymes GmbH, Darmstadt, Germany). The enzyme mixture was added in an enzyme-to-substrate ratio of 1:100 and incubated for 1 h at 37 °C with shaking at 180 rpm. The hydrolysis was stopped by heating the reaction mixture at 95 °C for 5 min. The hydrolysates obtained from proteolysis of IP-9 and IP-21 fractions were named IPH-9 and IPH-21, respectively. The hydrolysates IPH-9 and IPH-21 were lyophilised and stored at −20 °C until further use.

### 3.7. LC-MS and LC-MS/MS Analysis

LC-MS analysis of IP-9 and IP-21 fractions and LC-MS/MS analysis of IPH-9 and IPH-21 were carried out according to a previously described method [17,18] with some modifications. The IP samples were prepared at 0.2 mg/mL and the IPH samples at 0.5 mg/mL in injection buffer. Unlike IPH, IP fractions did not undergo a concentration phase using the PepMap100 microprecolumn. The separation step was performed on a PepMap100 C_18_ column (75 μm i.d. × 250 mm length, 3 μm particle size, 10 nm pore size; Dionex, Amsterdam, The Netherlands) at a flow rate of 300 nL/min. The injection volume was 0.5 μL for IP fractions and 1 μL for IPH. After a 5 min isocratic step of 5% solvent B (95% ACN + 0.08% HCOOH + 0.01% TFA), the elution gradient started with 5% of solvent B in solvent A (2% ACN + 0.08% HCOOH + 0.01% TFA) and reached 35% of solvent B over 35 min and then 85% in 2 more min before column re-equilibration. MS and MS/MS mass spectra were acquired in the same conditions, except that after the fragmentation of an ion, ions with the same m/z ratio were excluded for 20 s. Proteins and peptides were identified using LMD-9 and CNRZ-21N proteins FASTA files. Results were interpreted against the Uniprot database https://www.uniprot.org/, accessed on 15 November 2021. The protein modifications retained were serine phosphorylation and methionine oxidation.

### 3.8. Anti-Inflammatory Assay, Cell Cytotoxicity, ELISA and Western Blotting

The anti-inflammatory activities of IPH-9 and IPH-21 were evaluated in THP-1 macrophages (ECACC, Sigma-Aldrich, Saint Quentin Fallavier, France) according to Allouche et al. [17,18]. Briefly, THP-1 cells were maintained in Roswell Park Memorial Institute medium (RPMI 1640, Sigma-Aldrich, Saint Quentin Fallavier, France) supplemented with 10% heat-inactivated foetal bovine serum (FBS), 1% sodium pyruvate and 1% penicillin/streptomycin solution at 37 °C in a humidified 5% CO_2_ atmosphere. Culture medium was changed every 48 h. For anti-inflammatory experiments, THP-1 monocyte cells (8 × 10^5^ cells/mL) in complete culture media containing 20 ng/mL phorbol 12-myristate 13-acetate (PMA; Sigma-Aldrich, Saint Quentin Fallavier, France) were seeded in a 24-well plate (0.5 mL/well). These monocytic cells were differentiated into mature macrophage-like state after 48 h of PMA stimulation. Following PMA removal, the differentiated cells were washed twice with pre-warmed PBS and were again incubated for another 24 h with 0.5 mL cell culture medium devoid of PMA and antibiotics. Then, THP-1 macrophages were co-stimulated with 50 ng/mL lipopolysaccharide (LPS) and two concentrations of IPH-9 or IPH-21 (0.5 and 1.0 mg/mL) for 3 h. Cells treated with LPS alone were used as the reference control. For a positive control, THP-1 macrophages were co-incubated with 25 µM dexamethasone (DEX) solution (Sigma-Aldrich, Saint Quentin Fallavier, France) and LPS under the same conditions. After 3 h co-treatment, the supernatant was collected from each well, centrifuged at 14,000× *g* for 5 min at 4 °C and stored at −80 °C until they were used for IL-1β, IL-8 and TNF-α cytokines quantification by ELISA kits (Thermo Fisher Scientific, Villebon-sur-Yvette, France) following the manufacturer’s instructions. Whereas, cells were washed twice with PBS and then lysed to perform Western blot analysis, as described previously [17,18]. The cytotoxicity of IPH-9 and IPH-21 was assessed using an LDH cytotoxicity detection kit (Sigma-Aldrich, Saint Quentin Fallavier, France) according to Allouche et al. [18].

### 3.9. In Silico Anti-Inflammatory Prediction

In silico, the evaluation of the anti-inflammatory potential of the peptides identified in IPH-9 and IPH-21 after LC/MS-MS analysis was carried out using the database PreAIP (http://kurata14.bio.kyutech.ac.jp/PreAIP/, accessed on 15 January 2022). Peptide sequence in the FASTA format was used for the analyses.

### 3.10. Statistical Analysis

ELISA and Western blot results were subjected to one-way analysis of variance (ANOVA) followed by Dunnett’s test to find the statistical differences between samples. The statistical analyses were performed using GraphPad Prism 9.0.2 (GraphPad Software, San Diego, CA, USA), and the results were represented as the mean ± standard error of the mean (SEM). A *p*-value of <0.05 was considered statistically significant.

## 4. Conclusions

To our knowledge, this is the first study evaluating the anti-inflammatory activity of peptides derived from intracellular proteins of *S. thermophilus* strains. The hydrolysates obtained after Corolase PP hydrolysis of these intracellular proteins could be considered as postbiotics with potential anti-inflammatory effects for human health, which could make them promising bioactive ingredients for the development of functional foods aimed at preventing pathologies with an inflammatory component. These postbiotics, which are likely to be released in the consumer’s intestinal tract, could participate in the overall anti-inflammatory effect of this bacterium that was previously demonstrated with some strains of *S. thermophilus*.

## Figures and Tables

**Table 1:** Proteins identified by LC-MS/MS after Corolase PP hydrolysis of 3–10 kDa intracellular protein (IP) fractions of *S. thermophilus* LMD-9 (IP-9) and CNRZ-21N (IP-21).

			LMD-9			CNRZ-21N		
Protein Id	Description	pI *	MW * (kDa)	Nb Specific Sequences Identified	Coverage (%)	MW * (kDa)	Nb Specific Sequences Identified	Coverage (%)
STER_1896|ID:1900559|rplX|	50S ribosomal subunit protein L24	9.91	10.83	78	99.02	10.86	126	99.02
STER_1963|ID:1899676|	conserved protein of unknown function	6.29	7.07	67	98.53			
STER_1889|ID:1900552|rpmD|	50S ribosomal subunit protein L30	10.12	6.39	45	96.72	6.39	62	98.36
STER_1243|ID:1900052|	Phosphocarrier protein HPr (Histidine-containing protein)	4.88	8.91	47	93.18	8.91	95	98.86
STER_1892|ID:1900555|rplF|	50S ribosomal subunit protein L6	9.47	19.44	33	83.80	19.44	41	93.86
STER_0456|ID:1899076|rpmA|	50S ribosomal subunit protein L27	10.33	10.31	28	64.95	10.31	45	69.07
STER_1317|ID:1899536|	conserved protein of unknown function	8.86	6.81	29	98.46	6.81	31	98.46
STER_1954|ID:1899671|rpmG|	50S ribosomal subunit protein L33	10.35	5.92	26	80.00	5.92	37	98.00
STER_1764|ID:1900458|rpsL|	30S ribosomal subunit protein S12	11.66	15.05	24	66.67	15.05	30	79.71
STER_0850|ID:1899831|rpsT|	30S ribosomal subunit protein S20	10.68	8.40	24	67.09	8.40	49	83.54
STER_1175|ID:1899990|hupB|	HU, DNA-binding transcriptional regulator, beta subunit	9.05	9.60	22	85.87	9.60	24	85.87
STER_0128|ID:1898817|rpsI|	30S ribosomal subunit protein S9	10.67	14.23	21	53.44	14.23	11	63.36
STER_0541|ID:1899153|	protein of unknown function	6.85	9.77	20	55.81			
STER_1888|ID:1900551|rplO|	50S ribosomal subunit protein L15	10.50	15.52	17	53.74	15.54	16	65.99
STER_1953|ID:1899670|rpmF|	50S ribosomal subunit protein L32	10.49	6.78	18	57.38	6.78	23	59.02
STER_1899|ID:1900562|rpmC|	50S ribosomal subunit protein L29	9.35	7.90	17	91.30	7.90	15	98.55
STER_1141|ID:1899514|	exported protein of unknown function	10.84	6.26	18	69.49			
STER_1936|ID:1899661|	50S ribosomal protein L28	11.21	6.91	16	60.32	6.91	25	60.32
STER_0252|ID:1898920|groS|	Cpn10 chaperonin GroES, small subunit of GroESL	4.56	9.91	15	80.21			
STER_0787|ID:1899802|ykgM|	putative ribosomal protein	8.00	9.32	15	74.07			
STER_0455|ID:1899075|rplU|	50S ribosomal subunit protein L21	9.86	11.18	15	41.91	11.18	17	75.24
STER_1904|ID:1900567|rplB|	50S ribosomal subunit protein L2	10.57	29.91	13	51.80			
STER_1451|ID:1900209|	30S ribosomal protein S21 (BS-B)	11.29	6.91	12	59.32	6.92	9	59,32
STER_1902|ID:1900565|rplV|	50S ribosomal subunit protein L22	11.02	12.41	9	53.91	12.41	11	54.78
STER_1882|ID:1900545|rpsK|	30S ribosomal subunit protein S11	11.44	13.36	11	53.91	13.36	10	67.19
STER_1891|ID:1900554|rplR|	50S ribosomal subunit protein L18	9.84	12.88	11	43.70			
STER_1506|ID:1900257|rpsP|	30S ribosomal subunit protein S16	10.05	10.40	9	65.93	10.40	13	89.01
STER_0568|ID:1899173|rplL|	50S ribosomal subunit protein L7/L12	4.42	12.35	6	51.22			
STER_1798|ID:1900486|rplK|	50S ribosomal subunit protein L11	9.47	14.79	6	51.41	14.79	21	67.61
STER_1885|ID:1900548|infA|	translation initiation factor IF-1	8.06	8.24	4	57.53	8.24	4	61.64
STER_1903|ID:1900566|rpsS|	30S ribosomal subunit protein S19	10.04	10.54	5	65.59	10.61	5	63.44
STHERMOCNRZ21N_v1_10599|ID:59659749|	conserved protein of unknown function	6.85				9.77	26	60.47
STHERMOCNRZ21N_v1_30315|ID:59660455|	conserved exported protein of unknown function	11.45				6.31	23	83.05
STHERMOCNRZ21N_v1_30185|ID:59660325|	conserved protein of unknown function	6.29				7.13	19	97.06
STHERMOCNRZ21N_v1_30920|ID:59661060|rpsR|	ribosomal protein S18	10.63				9.26	10	46.25
STHERMOCNRZ21N_v1_30627|ID:59660767|rpsU|	ribosomal protein S21	11.29				6.92	9	59.32
STHERMOCNRZ21N_v1_10341|ID:59659491|rpmEB|	ribosomal protein L31	8.89				10.36	9	67.42
STHERMOCNRZ21N_v1_31101|ID:59661241|rpsNA|	ribosomal protein S14	10.61				7.05	7	53.23
STHERMOCNRZ21N_v1_10428|ID:59659578|	conserved protein of unknown function	9.10				4.92	4	68.18

* MW and pI were calculated using the Compute pI/Mw tool from Expasy.

## Data Availability

Data are contained within the article.
